# Innovative Natural Functional Ingredients from Olive and Citrus Extracts in Spanish-Type Dry-Cured Sausage “Fuet”

**DOI:** 10.3390/antiox10020180

**Published:** 2021-01-27

**Authors:** Lorena Martínez Zamora, Rocío Peñalver, Gaspar Ros, Gema Nieto

**Affiliations:** Department of Food Technology, Food Science and Nutrition, Faculty of Veterinary Sciences, Regional Campus of International Excellence “Campus Mare Nostrum”, University of Murcia, Espinardo, 30100 Murcia, Spain; lorena.martinez23@um.es (L.M.Z.); rocio.penalver@um.es (R.P.); gros@um.es (G.R.)

**Keywords:** antioxidants, dry-cured, sausage, hydroxytyrosol, citrus, functional, clean label

## Abstract

The main objective of the present study was to evaluate the antioxidant capacity of hydroxytyrosol derived from synthetic (HT_s_) and organic (HT_o_) sources, and citrus (C) extract, by incorporating them in a dry-cured meat product: fuet. Firstly, antioxidant extracts were tested in an oxidized pork meat model system, avoiding by 100% the protein oxidation against AAPH and AMVN. After that, four batches of fuet were made, namely Control, HT_s_, HT_o_, and C, which incorporated antioxidant extracts as substitutes of synthetic additives. A hundred-day shelf-life study was carried out. The incorporation of phenolic extracts neither affected proximal composition, nor ripening process (airing losses, a_w_, and pH), nor color development. However, the incorporation of HT increased Fe, Mn, and Si mineral content. At the same time, HT extracts inhibited lipid and protein oxidation and microbiological growth by 50%. Regarding sensory analysis, HT_o_ was the most unpalatable (extract flavor apparition), while HT_s_ and C samples were equally accepted as the Control sample. In addition, HT fuet samples showed two-fold higher antioxidant activity and total phenolic content than the Control sample. In conclusion, the use of HT_s_ in dry-cured sausages was demonstrated to be the best option to the development of clean label meat products, with promising antioxidant properties and the best standards of quality and acceptability.

## 1. Introduction

Meat and meat products constitute a food group of great nutritional importance due to their high content of protein, essential amino acids, Fe, Zn, and B-complex vitamins. However, the wide use of synthetic additives as preservatives in meat products poses a risk to human health due to their potential harmful effects after a long and continued consumption [1].

The main substrate that initiates the lipid peroxidation reactions in the meat is fat, which is also an essential ingredient for its organoleptic quality [2]. Due to its composition, meat is prone to oxidation reactions that result in a loss of quality during storage, as a consequence of the oxidation of lipids, proteins, and pigments such as myoglobin, which reduce the shelf life of the product by exerting a detrimental effect on color, flavor, texture, and nutritional value [3]. For this reason, the food industry has used synthetic antioxidants to control oxidation reactions, but, due to toxicity concerns, research is being conducted on the use of natural antioxidants that do not induce these harmful effects on health [1].

One of the main strategies to develop clean label meat products is the replacement of these additives by products of natural origin, such as plant extracts that are rich in polyphenols and flavonoids, and therefore have antioxidant and antimicrobial properties. These natural antioxidants act by preventing the formation of reactive oxygen species in the food and decreasing the oxidative processes in vivo, exerting a protective effect against many diseases [3]. Advances in this field can give meat industry the opportunity to produce clean label products free of synthetic antioxidants and preservatives [4].

This new trend has emerged because consumers are largely demanding healthy products that provide beneficial effects for human health by increasing their nutritional value. This fact is leading the food industry to search for compounds that enable the development of functional foods with added value [5].

One of these compounds is hydroxytyrosol, a by-product from olive oil production that could replace synthetic antioxidants, since it has a high antioxidant capacity and numerous beneficial effects on health. This phytochemical can be obtained from olive oil (organic origin), or from the hydrolysis of oleuropein (synthetic origin) [6]. In this way, the hydroxytyrosol of chemical synthesis has a purity higher than 90% and is able to alleviate the undesirable own flavors from olive tree and derivatives, while organic extract of hydroxytyrosol has a maximum purity of 20%, and its application also provides many other compounds that can decrease the sensory quality of the food product.

In this sense, the preservative capacity of hydroxytyrosol can be compared with known antioxidant extracts, such as hesperidin from citrus species, which has been demonstrated to act as antioxidant and antimicrobial in fresh and dry-cured pork meat products [7,8,9,10].

Dry-cured meat products are ripened during a period in which dehydration and microbiological changes are produced. These changes allow the development of their characteristic odor and flavor. This flavor development is also due to the incorporation of different additives, spices, and condiments. Fuet is a traditional fermented sausage which is made with minced pork fat and meat seasoned with salt and pepper, among other spices. Starter cultures are also incorporated into its formula, in order to control the presence of microorganisms that can alter their quality. Finally, it is stuffed into a thin pork casing with a caliber of 34–36 mm.

This product is typical from the Catalan gastronomy, and several researchers have included natural extracts and new technologies to prolong the shelf-life of these kind of products (three months, approximately). For example, Hospital et al. [11] studied the toxigenesis of *Clostridium botulinum* in nitrate and nitrite-reduced dry fermented sausages (“salchichón” and “fuet”) for 28 days. In a similar product, Lorenzo et al. [12] demonstrated that grape seed and chestnut extracts were more effective than synthetic antioxidants (BHT) against lipid oxidation in dry-cured “chorizo”. Moreover, the incorporation of rosemary and citric extracts into the formulation of clean-label dry-cured meat products has demonstrated to be effective against microbiological growth and the production of volatile compounds in Spanish “chorizo” [7,13] and in “Cinta Senese” dry-fermented sausages [9,10]. Additionally, chitosan essential oil has improved the microbiological, physicochemical, and sensory characteristics of Turkish fermented sausages (sucuk) [14].

The main objective of the present study was to know the preservative activity of hydroxytyrosol extracts from different sources and citric extract in a dry-cured sausage: traditional “fuet”. For that, lipid and protein oxidative damage, microbiological growth, and sensory perception were measured with the aim of evaluating the quality of a cured meat product enriched with natural extracts with potential health benefits. For that, the natural extracts used were previously tested in a pork meat model system, and the antioxidant activity of the final product (fuet) was also tested.

## 2. Materials and Methods

### 2.1. Preservative Extracts

Hydroxytyrosol (HT_o_), obtained from vegetation waters of olive (Olea europaea) with 7.26% pure bioactive compound and citric (*Citrus sinensis* L.) (C) with 55.1% hesperidin were supplied by Nutrafur-Frutarom, S. A. (Alcantarilla, Murcia, Spain). The antioxidant activity of these extracts was compared to hydroxytyrosol (HT_s_), which was synthetized by acid hydrolysis of oleuropein from DOPAC. In this way, this extract had a purity of 99.2% hydroxytyrosol and 0.3% hydroxytyrosol acetate. HT_s_ was supplied by Seprox Biotech, S. L. (Fuente Álamo, Murcia, Spain).

### 2.2. Protein Oxidation in an Oxidized Pork Meat Model System

#### 2.2.1. Oxidation Pork Meat Model System

A total of 500 g of pork loin was purchased from a local supermarket (Hipercor, S.A., Murcia, Spain). Fat was removed and meat was minced, using a grinder (12 °C, 2 min, 500 rpm). Minced meat was vacuum-packed in bags of 20 g and stored at −18 °C until analysis. Then, 1.5 g of minced meat was homogenized in 12.5 mL of 0.05 M MES buffer, pH =5.8, together with 200 ppm of extracts (HT_o_, HT_s_, and C). During homogenization using an Ultra Turrax T25 at 11,500 rpm for 30 s, samples were kept on ice, to minimize the oxidation rate. After that, the azo-initiators, 2,2′-azobis (2-amidinopropane) dihydrochloride (AAPH) diluted in Milli-Q water (0.54 mM) or 2,2′-azobis (2,4-dimethylvaleronitrile (AMVN) diluted in 99.9% EtOH (3 mM), were added as hydrophilic oxidation initiator (OX_AAPH_), or lipophilic oxidation initiator (OX_AMVN_), respectively. Immediately after addition of the azo-initiators, samples were placed in a water bath, under agitation, at 37 °C, for 200 min to oxidize the meat model system. The control used with no oxidizers was called Control NO-OX. After oxidation, thiol groups were quantified.

#### 2.2.2. Thiol Analysis

The thiol groups’ concentration is related to the protein oxidation and was determined after derivatization by 5,5′-dithiobis (2-nitrobenzoic acid) (DTNB) [15]. The method carried out to the analysis of thiol groups’ concentration was previously described by Martínez et al. [8].

### 2.3. Elaboration of Cured Meat Product: Fuet

The fuet samples (N = 160) were divided into four different batches of fuet (40 samples per batch, 5 samples of each batch per day of analysis). For that, pork minced meat and fat were purchased in a local supermarket, Hipercor, S.A. (Murcia, Spain). The “commercial mix” used for the preparation of Control fuet samples contains the following ingredients: salt, dextrin, dextrose, stabilizer: sodium phosphate (E-451), spices and spice extract, flavor, antioxidants: sodium ascorbate (E-301) and sodium citrate (E-331), preservatives: potassium nitrate (E-252) and sodium nitrite (E-250). This “commercial mix” was provided by Catalina Food Solutions S.L. (El Palmar, Murcia, Spain). The same “commercial mix” with no sodium ascorbate (E-301) and sodium citrate (E-331) was used for the reformulated samples. Microsan-R (a commercial starter culture) composed of *Pediococcus* (50%), *Staphylococcus xylosus* (25%), and *Staphylococcus carnosus* (25%) was also purchased from Catalina Food Solutions S.L. (El Palmar, Murcia, Spain) and used to enable fermentation. The lyophilized culture was rehydrated for 8 h prior to product manufacture (50 g in 750 mL in Milli-Q water) and sown in the mass at 6 × 107 CFU/g.

The meat was chopped and mixed with the rest of ingredients and extracts at concentrations described in Table 1. Then, the paste was stuffed into swine casing, using an automatic stuffer (Silvercrest ^®^ kitchen tools, Barcelona, Spain). The natural pork casing (40–42 mm ø) was previously desalted and washed with Milli-Q water. Each sample weighed 250 g, approximately. After elaboration, the fuet samples were labeled, weighed, and placed in an air-drying chamber Binder 115 redLine RI (Tuttlingen, Germany) set at 22 ± 1 °C and 90 ± 5% RH for two days. After the 2nd day of storage, temperature and humidity were adjusted to 14 ± 1 °C and 70 ± 5% RH for 12 days. Analysis was carried out in two phases: during ripening at 0, 7, and 14; and after curation process for 150 days, when samples were stored into plastic bags in aerobic conditions at 5 ± 1 °C, 65 ± 5% RH. Analysis during the refrigerated storage was carried out on days 21, 35, 50, and 100 after elaboration.

### 2.4. Proximal Composition

Fuet samples were analyzed for their moisture, ash, lipid, and total protein contents according to AOAC methods [16] on the same day of elaboration. The mineral concentrations of fuet samples were measured by plasma spectroscopy (ICP-OES) using an ICAP THERMO DUO 6500 computer.

### 2.5. Physicochemical Determinations during Ripening Process

Samples were weighed to measure losses because of airing during the ripening process, which was calculated as percent by the difference of weight from day 0 to 21. pH was measured using Crison GLP21 equipment (Crison Instruments S.A., Barcelona, Spain). Water activity (aw) was measured during the ripening process using the Lab Partner-aw (Novasina, A.G., Lachen, Switzerland). Analysis of airborne losses, pH, and water activity were carried out at days 0, 7, 14, 21, 35, 50, and 100 from elaboration.

### 2.6. Oxidation Shelf-Life Study for 100 Days

A Konica Minolta CR 400 colorimeter was used for color evaluation. Each day of analysis was calibrated with a standardized plate. This instrument uses the CIELab system, which provides data on lightness (L *), chroma coordinates a * (green–red chromaticity) and b * (blue–yellow chromaticity), chroma (C *), and hue (h). The measurements were made in triplicate on different parts of each sample at days 0, 7, 14, 21, 35, 50, and 100 after elaboration.

The thiol groups concentration is related to the protein oxidation and was determined after derivatization by 5,5′-dithiobis (2-nitrobenzoic acid) (DTNB) [15]. The method carried out to the analysis of thiol groups concentration in fuet samples was previously described by Martínez, Ros and Nieto [17]. The measurements were made in triplicate at days 0, 7, 14, 21, 35, 50, and 100 after elaboration.

Lipid oxidation was related to thiobarbituric acid reactive substances (TBARs) content, which were measured following the method described by Martínez, Ros and Nieto [17]. The TBARs value was reported in mg MDA/kg sample. The measurements were made in triplicate at days 0, 7, 14, 21, 35, 50, and 100 after elaboration.

### 2.7. Microbiological Analysis

Analysis of total vial count (TVC), total coliform count (TCC), and *Escherichia coli* were performed on day 21 of the study. All the samples were analyzed in triplicate, and the counts were expressed as colony forming units per gram (CFU/g). Samples were prepared in a horizontal laminar flow cabinet (Telstar, BIO-II-A, Spain) sterilized by UV irradiation. All media (PCA to determine TVC and Rapid E. Coli to determine TCC and *E. coli*) were prepared and sterilized at 121 °C, for 20 min, according to product indications. Peptone water (OXOID, Ltd. CM0087 Basingstoke, Hampshire, UK) was used to make the dilutions. After mass seeding, plates were incubated for 48 h, at 37 °C, for TVC; 24 h, at 37 °C, for TCC; and 48 h, at 45 °C, for *E. coli*.

### 2.8. Sensory Analysis

The tasting room for sensory evaluation was air-conditioned and free of disturbing factors. The fuet samples were cut in slices of 3–5 mm thickness. Sensory analysis was carried out at 21 days after elaboration.

Previously, ten panelists were trained according to the ISO guide [18] to carry out a quantitative descriptive sensory analysis. In total, there were two training sessions, where different descriptors related to the odor and flavor of samples were quantified and identified by the panelists. These attributes were evaluated using an intensity scale from 1 (minimum: undetectable) to 4 (maximum score: very intense). Samples were coded with random three digits and were presented individually to the panelists. Mineral water and stick breads were provided for mouth rinsing between samples. The attributes measured for the color, odor, and taste characteristics were as follows: “Red Color”, “Brown Color”, “Extract Color”, “Brightness”, “Own Odor”, “Cured Odor”, “Rancid Odor”, “Extract Odor”, “Own Flavor”, “Cured Flavor”, “Extract Flavor”, “Rancid Flavor”, “Hardness”, “Chewability”, “Juiciness”, and “Granularity”. “Acceptability” of fuet samples was measured by a panel of twenty consumers following an intensity scale from 1 (minimum: I do not like it) to 4 (maximum: I like it very much/I would by it) [19].

### 2.9. Antioxidant Capacity of Cured Meat Product

Previously to analysis, extracts of each fuet sample were obtained. For that, 2 g of sample was placed in plastic tubes with 10 mL of ethanol/Milli-Q water (25/75). This solution was mixed during 1 h, at 500 rpm, in an ice bath, and centrifuged at 3500 rpm, at 4 °C, for 4 min. The supernatant was filtered (0.2 µm) and kept at −80 °C until analysis [20]. Extractions were carried out with samples at day 21 after elaboration.

The total phenolic content (TPC) was determined quantitatively by using the Folin–Ciocalteu reagent and gallic acid as the standard [21]. The TPC was expressed as mg gallic acid equivalents (GAEs) per g of extract. 

The ferric-ion-reducing antioxidant power assay (FRAP) was also performed [22]. The FRAP reagent was daily prepared with 20 mL of 300 mmol/L acetate buffer, pH = 3.6, 2 mL 20 mmol/L FeCl_3_ 6 H_2_O, and 2 mL 10 mmol/L TPTZ (2,4,6-tripyridyl-s-triazine) in 40 mmol/L HCl. Trolox standard solutions at different concentrations were used as standard curve, in order to compare obtained results of the samples. The antioxidant power was expressed as µM Trolox equivalents (TE) per g extract.

The hydrophilic antioxidant capacity was measured by using the ORAC (Oxygen Radical Absorbance Capacity) method [23]. For that, the method described by González et al. [20] was carried out. All dilution samples were prepared in triplicate. The antioxidant activity of the sample was expressed as µM of Trolox equivalents (TE) per 100 g of sample. 

### 2.10. Statistical Analysis

Data were analyzed with the statistical package SPSS 15.0 (Statistical Package for the Social Science for Window (IBM, Armonk, NY, USA). The obtained results of in vitro antioxidant capacity and shelf-life study were analyzed, using ANOVA. The obtained results of the sensory evaluations were analyzed, using ANOVA, considering the effect of panelist and replicate. A value of *p* < 0.05 was considered statistically significant. The Scheefe test was applied, to test differences among groups.

## 3. Results

### 3.1. Protein Oxidation in a Pork Meat Model System after Addition of Antioxidant Extracts

**Figure 1:**
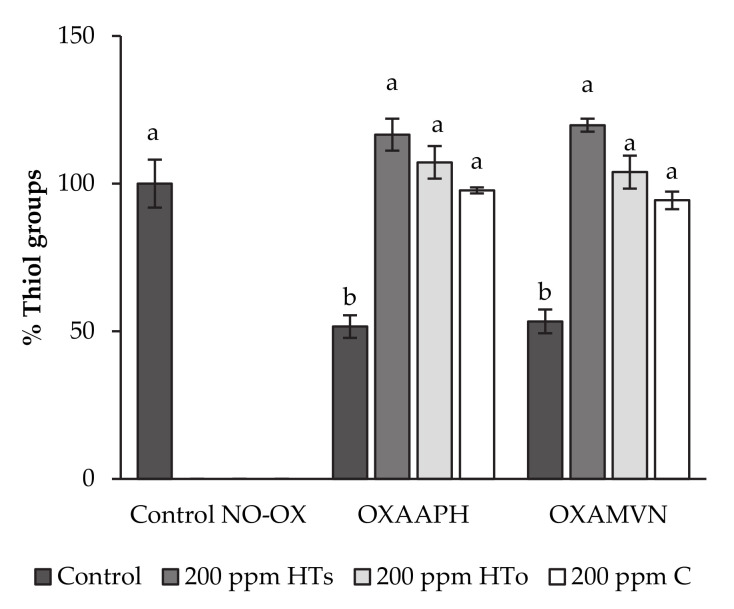
Percentage thiol groups in meat model systems oxidized by AAPH (OX_AAPH_) or AMVN (OX_AMVN_) after addition of phenolic extracts (HT_s_ (200 ppm), HT_o_ (200 ppm), and C (200 ppm) relative to a control meat model system with no oxidant (Control NO-OX). Different letters (a and b) indicate significant differences among samples (*p* < 0.05).

Figure 1 shows the relative concentration of thiol groups in an oxidized pork meat model system after addition of examined antioxidant extracts. 

The concentration of protein thiols in the control pork meat model system (Control NO-OX) was 46.7 ± 3.8 mmol/mg protein (100%). Subjecting the meat model system to oxidation by the hydrophilic initiator (OXAAPH) or the lipophilic initiator (OXAMVN) resulted in thiol concentrations of 23.1 ± 1.9 mmol/mg protein and 24.5 ± 2.0 mmol/mg protein, respectively (50% approximately) (Figure 1).

As indicated, the incorporation of antioxidant extracts (HT_s_, HT_o_, and C) completely inhibited (100%) the oxidation produced by AAPH and AMVN as oxidizers agents. In this way, no significant differences can be observed between Control NO-OX and oxidized pork meat model systems (OXAAPH and OXAMVN) that incorporated HT_s_, HT_o_, and C.

### 3.2. Proximal Composition Fuet Samples

Once fuet samples were made, proximate composition and mineral content was determined. Obtained results are shown in Table 2. As demonstrated, there were no significant differences among samples regarding proximate composition. In this manner, studied samples presented 32.1% moisture, 9.9% ash, 27.1% protein, and 27.1% lipid content, approximately. In contrast, significant differences (*p* < 0.05) were found in Fe, Mn, and Si content among the studied reformulations. In fact, all analyses followed the same behavior regarding mineral content. Therefore, the highest values were obtained by HT_o_ and HT_s_, followed by C and the Control sample. 

### 3.3. Physicochemical Results during Ripening Process

Table 3 shows the physical changes of fuet samples during ripening process.

As observed, there were no significant differences among reformulated samples with regard to airing losses through ripening process. In this way, losses of 50% (*p* < 0.05) were noticed during ripening time (from elaboration to 14th day). After this moment, airing losses were maintained during the shelf-life study with no significant differences among samples and day of analysis (data not shown).

Water activity (a_w_) is an essential parameter to control during curation process and it is directly related to airing losses. As the airing losses increased, the water activity of the studied samples decreased (*p* < 0.05) from 0.9285 to 0.756, at day 14. This parameter is then stabilized until the end of the shelf-life study with no significant differences (data not shown). In Table 3, no significant differences are found among different samples; therefore, the incorporation of different antioxidant extracts did not alter these values compared to the Control sample.

Similarly, pH values were significantly decreased (*p* < 0.05) from 6.13, at day 0, to 5.36, at day 14 from elaboration, when, again, these values stabilized until the end of the shelf-study, at day 100 (data not shown). Furthermore, no significant differences were observed among the studied reformulations, which demonstrated that HT and C incorporation did not affect pH and curation process of fuet samples.

Table 4 shows the obtained results of the CIELab analysis from day 0 to day 100 of shelf-life study. As appreciated, there is only significant differences among samples with regard to luminosity (L *) (*p* < 0.05). In this way, HT_s_ was the only sample able to maintain the L * through the shelf-life study, even better than the Control sample, which could be observed on day 100 (*p* < 0.05). In contrast, studied samples did not present significant differences with regard to the rest of color the coordinates. Then, a * (redness coordinate) values presented a slightly increase from 10.1 to 18.2, at the end of the ripening, when again it decreases to 13.9. In contrast, b * (yellowness coordinate) values suffered a decrease from 14.1 to 7.5 during the shelf-life study. Similarly, values of C * (Chroma or saturation) and h (hue or tone) also suffered a decrease from 17.3 and 54.3 to 16.6 and 26.6, respectively.

**Figure 2:**
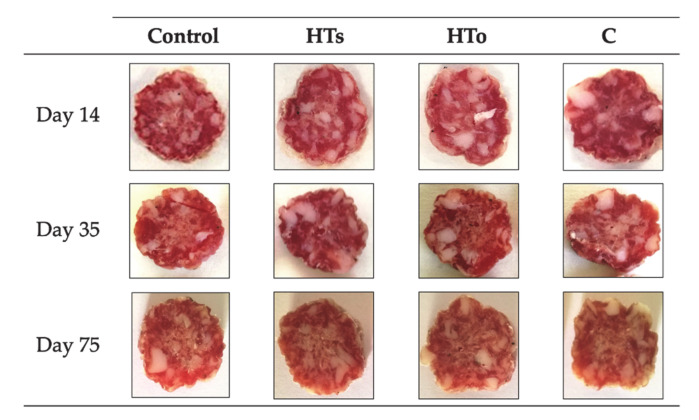
Photographs of reformulated fuet samples. HT_s_, samples enriched with synthetic Hydroxytyrosol; HT_o_, samples enriched with organic Hydroxytyrosol; C, samples enriched with citric.

These variations can be also visually observed in Figure 2, which shows the evolution of fuet samples from day 14 to 75. Then, the losses of L *, a * (redness tones), C *, and h can be observed when fuet samples reach the 75th day, as the color is much duller than at the beginning of the shelf-life study.

### 3.4. Oxidation Shelf-Life Study for 150 Days

Results of lipid and protein oxidation during the shelf-life study are shown in Table 5. As observed, regarding lipid oxidation, there were significant differences from the seventh day to the end of the refrigerated storage. In fact, HT_s_ showed an important decrease of the TBARs value in all the sampling-days, which demonstrated that HT extracts, particularly HT_s_ inhibited lipid degradation in a better way that synthetic preservatives used in the Control sample. Furthermore, at day 100 from elaboration, a reduction of 80% mg MDA/kg sample was shown by HT_s_ and HT_o_ regarding C and Control samples (*p* < 0.05).

In contrast, protein oxidation was less affected by the incorporation of antioxidant extracts, and a general decrease from 54.1 mmol thiol/mg protein, at day 0, to 7.8 mmol thiol/mg protein, at day 100, (85.6% less) was observed. Indeed, no significant differences were found among samples until 35th day from elaboration, when HT_s_ extract also demonstrated that can avoid thiol loss as well as synthetic preservatives (Control). 

### 3.5. Microbiological Content

Table 6 shows the microbiological content of fuet samples after 21 days from elaboration. 

As observed, HT_s_ reduced TVC and TCC by 90 and 44%, respectively, compared to the Control sample. Similarly, HT_o_ reduced TVC and TCC by 68% and 28%, respectively, compared to the Control sample (*p* < 0.05). In contrast, no significant differences were found between the C and Control samples. Therefore, HT demonstrated a reduction of microbiological growth, while C did not. Moreover, no significant differences were found regarding *E. Coli* content in different reformulations.

### 3.6. Sensory Quality

**Figure 3:**
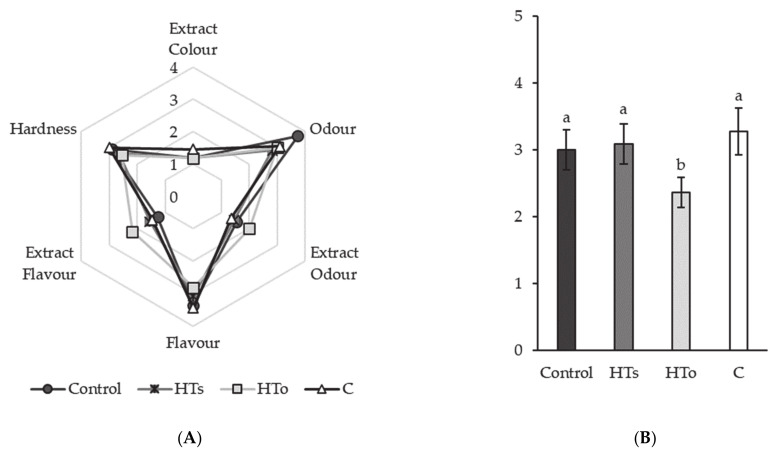
Sensory analysis (**A**) and acceptability (**B**) of fuet samples. HT_s_, samples enriched with synthetic Hydroxytyrosol; HT_o_, samples enriched with organic Hydroxytyrosol; C, samples enriched with citric. a, b Indicates significant differences among samples (*p* < 0.05).

Figure 3 shows the obtained results by sensory analysis. As observed, “odor”, “flavor”, and “texture” of reformulated samples were highly scored (3, 3.5, and 3, respectively) by panelists. In fact, no significant differences were found among different sensory parameters measured (Figure 3A). Nevertheless, HT_o_ obtained higher values of “Extract Flavor” (*p* < 0.05) (2.2 score) and “Extract Odor” (2 score) due to the origin of this extract (vegetation waters of olive). Indeed, this behavior was the reason of the decrease of the acceptability (Figure 3B) in HT_o_ samples by 25% (2.5 score) compared to the rest of fuet samples. Furthermore, the incorporation of HT_s_ and C to fuet samples did not affected sensory quality neither acceptability of the product, which was maintained to the highest levels (3.1 score). Hence, as preliminary results, it can be said that HT_s_ and C extracts acted as preservatives, while maintaining a good sensory perception of fuet samples.

### 3.7. Antioxidant Activity of Fuet Samples

Total phenolic content and antioxidant activity of reformulated fuet samples are shown in Table 7. For instance, it can be observed as samples made with antioxidant extracts (HT_s_, HT_o_, and C) presented two-fold higher total phenolic content than the Control sample (*p* < 0.05). Then, total antioxidant capacity of fuet samples was also increased by the incorporation of HT and C extracts by 36–28%, measured by ORAC_H_ method (*p* < 0.05). In contrast, measured by FRAP, HT_o_ and HT_s_ presented an antioxidant activity one and two times higher than the Control sample (*p* < 0.05), while the C sample did not show significant differences with regard to the Control. 

## 4. Discussion

The concentration of protein thiols in the control pork meat model system (Control NO-OX) was 46.7 ± 3.8 mmol/mg protein (100%), and is comparable to previous results reported by Martínez et al. [8] in a similar pork meat model system. In Figure 1, it is presented that HT_s_, HT_o_, and C extracts inhibited the protein oxidation, directly related with thiol loss, in presence of oxidizer agents (AAPH and AMVN) in the pork meat model system. In fact, HT is widely known as one of the most antioxidant compounds [4,24]. This bioactivity can be explained by its chemistry structure, which is formed by a phenol group and a hydroxyl group, which produce phenoxyl radicals in the presence of oxidizer agents. For that, this promising molecule, from HT_s_ (99% purity) and HT_o_ (7% purity), is able to avoid the thiol oxidation in the oxidized pork meat model, and, as a consequence, it could be a good protector against protein oxidation in processed meat products. Similarly, the antioxidant capacity of C is possibly attributed to its high concentration of hesperidin (55%). Actually, hesperidin is a bioflavonoid glycoside whose antioxidant capacity lies in the high number of hydroxyl groups. As well as HT extracts, the chemical structure of its main compound can justify the radical scavenging and antioxidant activity in the pork meat model system subjected to external oxidizers, such as AAPH and AMVN (Figure 1), protecting thiol groups from oxidative reactions and maintaining the thiol concentration at the same level that Control NO-OX. Obtained results by C agree with those obtained by us in a similar meat model system after incorporation of 500 ppm citrus extract [8].

With regards to the proximal composition, obtained results of fuet samples can be supported by Herrero et al. [25], who analyzed dry cured fermented sausages, such as chorizo, salchichón, salami, fuet, and mini-fuet. Herrero et al. [25] found 70% dry matter and 30% fat content, which are comparable with that of the present study (32% moisture and 27% fat content). As described, a significant increase (*p* < 0.05) was obtained with regard to Fe, Mn, and Si content of studied samples. These finding can be explained by the affinity of hydroxytyrosol to link to certain minerals, such as Fe in black olives, in order to catalyze the oxidation of gluconate Fe (II). Therefore, hydroxytyrosol may influence biological availability of some minerals [26]. Moreover, Martínez, Ros and Nieto [27] demonstrated that the presence of hydroxytyrosol in chicken meat emulsions increased the uptake of Fe by Caco-2 cells (in vitro). Due to this affinity between minerals and phenolics, an increase of Fe, Mn, or Si after hydroxytyrosol incorporation can be justified. 

Similar to the present study, obtained values of air losses, a_w_, and pH (Table 3) can be supported by Herrero et al. [25], who also showed comparable values in dry-cured fuet samples of different brands (a_w_ = 0.778 and pH = 5.42). In addition, similar conclusions were also reached by Hospital et al. [11] in nitrate and nitrite-reduced dry fermented Spanish sausages (“salchichón” and “fuet”) with regards to a_w_ values. 

Besides this, as it is widely known about dry-fermented sausages, starter cultures incorporated into dry-cured products, which are composed of lactic acid bacteria (*Pediococcus* (50%), *Staphylococcus xylosus* (25%), and *Staphylococcus carnosus* (25%), in this case), result in the fermentation of sugars, which produces a descent of pH values close to 5, in order to avoid the growth of pathogenic microorganisms, such as *Clostridium botulinum* [28]. Moreover, the antimicrobial effect of nitrites is higher at pH around 4.5 and 5.5. Therefore, all studied samples were within the optimal range of pathogenic microorganism inhibition by nitrites.

Table 4 shows obtained values of color development during ripening and refrigerated storage. The development of the characteristic reddish color (a *) of the fuet did not present any type of incidence, both in the Control sample and in those incorporating HT and C. In the case of C sample, a decrease in the development of the reddish color (a *) (from day 35 to the end of the study) showing a paler appearance, while no effects on variation of red color during storage were observed after incorporation of HT and C, which was also noticeable in Figure 2. The color is one of the most decisive aspects in the process of choosing a cured sausage and the use of hydroxytyrosol does not seem to alter its development. These results agree with that of Chaves-López et al. [29] and Nieto et al. [30], where the addition of hydroxytyrosol maintained and increased the appearance of the reddish color in pork fermented sausages and chicken sausages, respectively.

Regarding the results showed in Table 5, TBARs’ values were kept below the limit of 1 mg MDA/kg of product, from which rancid taste is generated in the product. Then, this rancid taste did not develop in the first 50 days of analysis. However, the Control and C samples exceeded this value at day 100 of the shelf-life study, while HT extracts preserved fuet samples even after this date (*p* < 0.05). In agree with this fact, Cofrades et al. [31] showed as the addition of 100 ppm of hydroxytyrosol reduced lipid oxidation similarly to BHA/BHT in both Frankfurt sausages and cooked meat. Moreover, Muíño et al. [5] demonstrated that the addition of 100, 200, and 400 ppm of hydroxytyrosol reduced lipid oxidation in lamb pies in a similar way to the Control sample made with synthetic preservatives. Moreover, our previous researcher has demonstrated the reduction of lipid oxidation after the incorporation of hydroxytyrosol extracts in chicken sausages [30], chicken nuggets [17], and fish patties [32]. Therefore, the antioxidant capacity of hydroxytyrosol in the protein matrix has been repeatedly demonstrated and it justifies the results presented in the present study.

Moreover, Table 5 shows obtained results of protein oxidation, which is directly related to concentration of free thiol groups [33]. The concentration of the Control sample at the beginning of the shelf-life study was 54.1 ± 3.2 mmol/mg protein, and no significant differences were found among studied samples at day 0. However, a great descent (50%) (*p* < 0.05) was produced from this moment to the end of the ripening process, at day 21. This fact occurs as a result of the protein oxidation produced when free thiols form bounds among proteins, which changes protein structure increasing the hardness of the product as a consequence of curation process. In this way, after one hundred days of shelf-life, the study’s HT_s_ samples did not present significant differences with regard to the Control sample, while HT_o_ and C slightly decreased the concentration regarding those samples. These results agree with antioxidant capacity of studied extracts after application in an oxidized pork meat model system (Figure 1). As previously described, HT and C extracts completely inhibited the action of AAPH and AMVN; therefore, it is clear not to have great differences among fuet samples after incorporation of antioxidants extracts (HT_s_, HT_o_, and C).

The microbial content of fuet samples measured at day 21 of the shelf-life study (Table 6) showed as HT_s_ inhibited by 90% TVC and by 44% TCC (*p* < 0.05). This behavior is widely known due to the antimicrobial activity of this compound. Otherwise, HT_o_ showed a lower antimicrobial capacity because of the purity of the organic extract (only 7% in comparison to 99% of HT_s_). The antibacterial and preservative action of hydroxytyrosol has previously demonstrated by several authors. For instance, Azaizeh et al. [34] showed as 400 μg/mL HT extract inhibited by 100% the growth of *Streptococcus pyogenes, Staphylococcus aureus, Escherichia coli,* and *Klebsiella pneumoniae*. In fact, this behavior has been also reported as food preservative in chicken nuggets for twelve months of frozen storage [17] and fish patties for eleven days of refrigerated storage [32,35] applied at 750 and 200 ppm, respectively.

Sensory description of reformulated fuet samples is shown in Figure 2. As previously described, acceptability of HT_o_ decreased by 25% (Figure 3B) due to “Extract Flavor”. This parameter obtained a higher perception by panelists (*p* < 0.05) (2.2 score) than the rest of reformulated samples (Figure 3A). However, the high acceptability reported by HT_s_ and C samples was remarkable, because there were no significant differences with regard to the Control sample. This fact can be justified by the purity of HT_s_, which did not contain phenolic derivatives from olive tree, as HT_o_ did, causing the bitter and strange taste that these fuet samples presented. This behavior was previously described by Nieto, Martínez and Ros [30], who reported strange flavors in chicken sausages after incorporation of 50 ppm hydroxytyrosol extracts obtained from olive leaf combined to 2.5% walnuts. In addition, there were not found significant differences among studied samples regarding the rest of evaluated parameters. Moreover, as observed in Figure 2, there were no apparent differences among the reformulated fuet, so from a visual point of view all the samples were also equally accepted.

Finally, the incorporation of HT and C extracts increased the total phenolic content in fuet samples (*p* < 0.05), which also improved the total antioxidant activity measured by ORAC and FRAP methods (Table 6). This fact demonstrated the functionality of studied fuets, because those parameters were increased even two times regarding the Control sample (*p* < 0.05). Obtained results can be supported by the rest of the assessed characteristics where the antioxidant and antimicrobial capacities of HT were demonstrated (Figure 1, Table 5 and Table 6). Additionally, the antioxidant activity of HT has been repeatedly demonstrated by several authors, as in vitro [36,37,38], as in vivo [39,40,41,42,43,44] or as a preserver in a food matrix [17,30,31,32,35,45,46,47]. For that, incorporation of this kind of products in a balanced Mediterranean Diet could report significant health benefits of consumers in comparison with usual manufactured meat products rich in synthetic additives.

## 5. Conclusions

The study of HT and C extracts demonstrated that they completely protect against protein oxidation in an oxidized pork meat model, which was subsequently demonstrated again after its application as a preservative in fuet samples. In fact, reformulated fuets did not present significant differences with regard to the Control sample during the ripening process. However, after that, HT_s_ showed the highest protection against lipid and protein oxidation, as well as against the microbiological growth. Furthermore, HT_o_ and C also reduced these phenomena at similar levels than the Control sample. Furthermore, HT_s_ did not alter sensory perception of fuet samples and significantly increased the antioxidant capacity and the total phenolic content of fuets. In conclusion, use of HT_s_ in dry-cured sausages demonstrated to be the best option among the tested products for the development of functional meat products, with promising antioxidant properties achieving the best standards of quality and sensory acceptability.

## Figures and Tables

**Table 1:** Formulation of fuet samples.

Ingredients	Samples			
	Control	HT_s_	HT_o_	C
Pork meat (g)	6300	6300	6300	6300
Pork fat (g)	2700	2700	2700	2700
Water (mL)	485	485	485	485
Commercial mix^®^ (g/kg)	75			
Basis^®^ (g/kg)		75	75	75
Ferment (mL)	180	180	180	180
Natural extracts (ppm):				
HT_s_		200		
HT_o_			200	
C				200

Commercial mix^®^ was composed of salt, dextrin, dextrose, stabilizer: sodium phosphate (E-451), spices and spice extract, flavor, antioxidants: sodium ascorbate (E-301) and sodium citrate (E-331), preservatives: potassium nitrate (E-252) and sodium nitrite (E-250). Basis^®^ was composed of: salt, dextrin, dextrose, stabilizer: sodium phosphate (E-451), spices and spice extract, flavor, preservatives: potassium nitrate (E-252) and sodium nitrite (E-250). HT_S_, synthetic hydroxytyrosol; HT_o_, organic hydroxytyrosol; C, citric.

**Table 2:** Proximate composition and mineral content of fuet samples.

	Proximate Composition (M ± SD)						
Samples	Moisture (%)	Ash (%)	Protein (%)	Lipid (%)	Fe	Mn	Si
**Control**	33.09 ± 3.15	9.31 ± 1.12	29.14 ± 2.13	26.83 ± 2.11	6.74 ± 0.06 ^c^	1.12 ± 0.00 ^c^	47.55 ± 1.01 ^d^
**HT_S_**	33.14 ± 1.19	9.59 ± 0.72	27.58 ± 0.74	27.98 ± 1.49	12.64 ± 0.36 ^a^	2.02 ± 0.05 ^a^	203.71 ± 9.04 ^b^
**HT_o_**	31.58 ± 0.81	9.95 ± 0.23	26.13 ± 0.31	26.76 ± 1.67	12.85 ± 0.11 ^a^	2.08 ± 0.16 ^a^	272.83 ± 9.79 ^a^
**C**	30.70 ± 1.25	10.81 ± 0.86	25.51 ± 2.02	26.66 ± 0.99	9.88 ± 0.09 ^b^	1.81 ± 0.02 ^b^	150.18 ± 2.65 ^c^

HT_s_, samples enriched with synthetic Hydroxytyrosol; HT_o_, samples enriched with organic Hydroxytyrosol; C, samples enriched with citrus. The mineral concentrations of fuet samples are expressed in mg/100 g. Only significant results are shown; ^a^, ^b^, ^c^, ^d^: different letters among data in the same column indicate significant differences among samples (*p* < 0.05).

**Table 3:** Physicochemical quality evolution of fuet samples during ripening process.

Samples				
*Days of Storage*	*Control*	*HT_s_*	*HT_o_*	*C*
**Airing losses (%)**				
Day 7	36.42 ± 1.02 ^b^	34.50 ± 2.03 ^b^	35.55 ± 2.08 ^b^	38.81 ± 3.00 ^c^
Day 14	50.23 ± 3.11 ^a^	47.74 ± 1.88 ^a^	49.95 ± 3.87 ^a^	48.52 ± 1.01 ^b^
Day 21	52.20 ± 2.23 ^a^	49.89 ± 3.20 ^a^	51.14 ± 1.34 ^a^	53.25 ± 0.99 ^a^
**a_w_**				
Day 0	0.929 ± 0.01 ^a^	0.928 ± 0.01 ^a^	0.928 ± 0.01 ^a^	0.929 ± 0.00 ^a^
Day 7	0.889 ± 0.00 ^b^	0.885 ± 0.05 ^b^	0.882 ± 0.03 ^b^	0.878 ± 0.02 ^b^
Day 14	0.785 ± 0.01 ^c^	0.777 ± 0.01 ^c^	0.745 ± 0.04 ^c^	0.752 ± 0.00 ^c^
Day 21	0.763 ± 0.02 ^c^	0.752 ± 0.00 ^c^	0.734 ± 0.01 ^c^	0.740 ± 0.01 ^c^
**pH**				
Day 0	6.15 ± 0.03 ^a^	6.18 ± 0.03 ^a^	6.03 ± 0.02 ^a^	6.16 ± 0.02 ^a^
Day 7	5.20 ± 0.02 ^b^	5.25 ± 0.00 ^b^	5.22 ± 0.01 ^b^	5.08 ± 0.00 ^b^
Day 14	5.40 ± 0.02 ^b^	5.31 ± 0.01 ^b^	5.31 ± 0.01 ^b^	5.28 ± 0.00 ^b^
Day 21	5.44 ± 0.05 ^b^	5.38 ± 0.04 ^b^	5.38 ± 0.02 ^b^	5.34 ± 0.02 ^b^

HT_s_, samples enriched with synthetic Hydroxytyrosol; HT_o_, samples enriched with organic Hydroxytyrosol; C, samples enriched with citric; ^a^, ^b^, ^c^: different letters among data in the same column indicate significant differences among days of analysis (*p* < 0.05).

**Table 4:** Color development during ripening and refrigerated storage of fuet samples.

		Control	HT_s_	HT_o_	C
**Day 0**	L*	62.09 ± 0.01 ^a w^	56.97 ± 0.03 ^b w^	57.54 ± 0.01 ^b w^	54.09 ± 0.04 ^c w^
	a*	9.86 ± 0.04 ^z^	9.69 ± 0.01 ^z^	10.52 ± 0.01 ^z^	10.30 ± 0.01 ^z^
	b*	14.06 ± 0.05 ^x^	14.07 ± 0.02 ^x^	14.78 ± 0.00 ^x^	13.34 ± 0.02 ^x^
	C*	17.17 ± 0.04 ^z^	17.08 ± 0.02 ^z^	18.14 ± 0.01 ^z^	16.86 ± 0.01 ^z^
	h	54.94 ± 0.19 ^x^	55.44 ± 0.03 ^x^	54.56 ± 0.02 ^x^	52.35 ± 0.06 ^x^
**Day 7**	L*	55.89 ± 0.04 ^c xw^	58.60 ± 0.05 ^a xw^	55.75 ± 0.11 ^c xw^	56.82 ± 0.10 ^b xw^
	a*	17.20 ± 0.29 ^y^	16.03 ± 0.01 ^y^	16.76 ± 0.07 ^y^	15.24 ± 0.06 ^y^
	b*	10.21 ± 0.21 ^y^	10.08 ± 0.01 ^y^	10.94 ± 0.01 ^y^	9.85 ± 0.06 ^y^
	C*	20.00 ± 0.36 ^yx^	18.94 ± 0.01 ^yx^	20.01 ± 0.06 ^yx^	18.15 ± 0.08 ^yx^
	h	30.70 ± 0.11 ^y^	32.16 ± 0.03 ^y^	33.14 ± 0.09 ^y^	32.89 ± 0.05 ^y^
**Day 14**	L*	57.75 ± 0.06 ^a yx^	55.31 ± 0.14 ^b yx^	54.19 ± 0.01 ^b yx^	50.48 ± 0.05 ^c yx^
	a*	14.81 ± 0.04 ^y^	16.72 ± 0.12 ^y^	14.57 ± 0.02 ^y^	17.57 ± 0.01 ^y^
	b*	8.6 ± 0.02 ^z^	9.86 ± 0.02 ^z^	8.14 ± 0.0 ^z^	9.51 ± 0.01 ^z^
	C*	17.13 ± 0.04 ^zy^	19.41 ± 0.12 ^zy^	16.69 ± 0.02 ^zy^	19.95 ± 0.05 ^zy^
	h	30.14 ± 0.02 ^z^	30.53 ± 0.12 ^z^	29.2 ± 0.02 ^z^	28.41 ± 0.02 ^z^
**Day 21**	L*	49.91 ± 0.01 ^c z^	51.18 ± 0.01 ^a z^	49.55 ± 0.01 ^c z^	50.29 ± 0.08 ^b z^
	a*	19.05 ± 0.01 ^x^	18.9 ± 0.01 ^x^	17.96 ± 0.02 ^x^	16.86 ± 0.03 ^x^
	b*	8.42 ± 0.01 ^z^	9.04 ± 0.01 ^z^	9.71 ± 0.01 ^z^	8.27 ± 0.01 ^z^
	C*	20.82 ± 0.01 ^x^	20.96 ± 0.01 ^x^	20.42 ± 0.02 ^x^	18.78 ± 0.04 ^x^
	h	23.86 ± 0.01 ^z^	25.56 ± 0.01 ^z^	28.41 ± 0.02 ^z^	26.12 ± 0.02 ^z^
**Day 35**	L*	54.45 ± 0.14 ^a z^	52.12 ± 0.19 ^b z^	48.64 ± 0.01 ^c z^	48.76 ± 0.02 ^c z^
	a*	15.98 ± 0.06 ^x^	17.87 ± 0.18 ^x^	19.30 ± 0.02 ^x^	18.73 ± 0.01 ^x^
	b*	7.86 ± 0.08 ^z^	9.50 ± 0.08 ^z^	10.33 ± 0.03 ^z^	9.59 ± 0.01 ^z^
	C*	17.81 ± 0.08 ^x^	20.24 ± 0.19 ^x^	21.90 ± 0.03 ^x^	21.05 ± 0.02 ^x^
	h	26.19 ± 0.16 ^z^	27.98 ± 0.06 ^z^	28.14 ± 0.03 ^z^	27.08 ± 0.01 ^z^
**Day 50**	L*	50.57 ± 0.38 ^c zy^	56.71 ± 0.10 ^a zy^	49.95 ± 0.05 ^c zy^	51.38 ± 0.01 ^b zy^
	a*	17.34 ± 0.04 ^y^	15.93 ± 0.02 ^y^	18.71 ± 0.01 ^y^	13.74 ± 0.01 ^y^
	b*	8.31 ± 0.01 ^z^	7.54 ± 0.03 ^z^	10.10 ± 0.01 ^z^	8.44 ± 0.01 ^z^
	C*	19.23 ± 0.04 ^zy^	17.62 ± 0.03 ^zy^	21.26 ± 0.01 ^zy^	16.13 ± 0.01 ^zy^
	h	25.62 ± 0.05 ^z^	25.35 ± 0.05 ^z^	28.37 ± 0.01 ^z^	31.57 ± 0.03 ^z^
**Day 100**	L*	46.32 ± 0.23 ^b zy^	52.17 ± 0.14 ^a zy^	45.75 ± 0.11 ^b zy^	47.18 ± 0.10 ^b zy^
	a*	15.34 ± 0.10 ^y^	13.83 ± 0.01 ^y^	15.33 ± 0.01 ^y^	11.24 ± 0.03 ^y^
	b*	7.10 ± 0.06 ^z^	6.94 ± 0.07 ^z^	9.08 ± 0.06 ^z^	6.99 ± 0.01 ^z^
	C*	18.83 ± 0.19 ^zy^	15.33 ± 0.25 ^zy^	20.26 ± 0.01 ^zy^	12.10 ± 0.31 ^zy^
	h	24.62 ± 0.45 ^z^	23.35 ± 0.15 ^z^	28.90 ± 0.57 ^z^	29.71 ± 0.29 ^z^

HT_s_, samples enriched with synthetic Hydroxytyrosol; HT_o_, samples enriched with organic Hydroxytyrosol; C, samples enriched with citric; ^a^, ^b^, ^c^: different letters among data in the same row indicate significant differences among samples (*p* < 0.05); ^w^, ^x^, ^y^, ^z^: different letters among data in the same column indicate significant differences among days of analysis (*p* < 0.05).

**Table 5:** Evolution lipid and protein oxidation of fuet samples for hundred days of shelf-life study.

Samples				
Time of Storage	Control	HT_s_	HT_o_	C
**Lipid Oxidation: TBARs (mg MDA/kg)**				
Day 0	0.08 ± 0.01	0.07 ± 0.01	0.07 ± 0.02	0.08 ± 0.01
Day 7	0.41 ± 0.09 ^b^	0.29 ± 0.06 ^c^	0.26 ± 0.01 ^c^	0.61 ± 0.12 ^a^
Day 14	0.50 ± 0.07 ^b^	0.39 ± 0.05 ^c^	0.34 ± 0.02 ^c^	0.70 ± 0.01 ^a^
Day 21	0.46 ± 0.07 ^b^	0.40 ± 0.06 ^c^	0.59 ± 0.15 ^a^	0.61 ± 0.17 ^a^
Day 35	0.42 ± 0.02 ^b^	0.28 ± 0.04 ^c^	0.34 ± 0.01 ^b^	0.34 ± 0.03 ^b^
Day 50	0.47 ± 0.04 ^b^	0.34 ± 0.05 ^c^	0.45 ± 0.03 ^b^	0.56 ± 0.03 ^a^
Day 100	3.04 ± 0.09 ^a^	0.62 ± 0.03 ^b^	0.68 ± 0.02 ^b^	3.37 ± 0.12 ^a^
**Protein Oxidation: Thiol Groups (mmol/mg protein)**				
Day 0	54.1 ± 3.2	51.4 ± 4.1	52.3 ± 3.1	53.6 ± 2.2
Day 7	35.0 ± 2.5 ^a^	25.6 ± 2.3 ^b^	27.5 ± 1.2 ^b^	24.3 ± 2.0 ^b^
Day 14	22.9 ± 0.8	24.4 ± 0.8	23.2 ± 0.9	24.0 ± 0.7
Day 21	18.9 ± 1.8	19.1 ± 1.2	21.0 ± 1.0	19.8 ± 1.1
Day 35	12.4 ± 1.0 ^b^	14.1 ± 1.0 ^b^	18.5 ± 0.5 ^a^	13.8 ± 1.4 ^b^
Day 50	9.9 ± 0.3 ^a^	6.9 ± 0.5 ^b^	7.7 ± 0.9 ^b^	8.8 ± 0.6 ^a^
Day 100	8.7 ± 0.5 ^a^	8.5 ± 0.2 ^a^	6.6 ± 0.5 ^b^	6.1 ± 0.3 ^b^

HT_s_, samples enriched with synthetic Hydroxytyrosol; HT_o_, samples enriched with organic Hydroxytyrosol; C, samples enriched with citric; ^a^, ^b^, ^c^, different letters among data in the same row indicate significant differences among samples (*p* < 0.05).

**Table 6:** Microbiological content (cfu/g) of fuet samples at day 21 of refrigerated storage in aerobic conditions.

Microorganism	Control	HT_s_	HT_o_	C
*TVC*	3.8 × 10^6^ ± 1.2 × 10^4 ab^	2.6 × 10^5^ ± 3.6 × 10^3 c^	1.2 × 10^6^ ± 6.4 × 10^4 b^	5.8 × 10^6^ ± 1.5 × 10^5 a^
*TCC*	90 ± 10 ^a^	50 ± 5 ^b^	65 ± 5 ^ab^	100 ± 10 ^a^
*E. Coli*	<10	<10	<10	<10

HT_s_, samples enriched with synthetic Hydroxytyrosol; HT_o_, samples enriched with organic Hydroxytyrosol; C, samples enriched with citric; TVC, Total Vial Count; TCC, Total Coliform Count; ^a^, ^b^, ^c^, different letters among data in the same row indicate significant differences among samples (*p* < 0.05).

**Table 7:** Total phenolic content (mg GAE kg^−1^) and antioxidant activity (µmol TE kg^−1^) of fuet samples.

Samples	Total Phenolic Content	Antioxidant Activity	
		ORAC_H_	FRAP
**Control**	105.5 ± 42.6 ^b^	2007.7 ± 99.1 ^b^	2411.8 ± 61.0 ^c^
**HT_S_**	206.4 ± 58.4 ^a^	2745.9 ± 85.7 ^a^	6403.7 ± 143.5 ^a^
**HT_o_**	203.0 ± 22.0 ^a^	2664.5 ± 153.8 ^a^	5341.6 ± 499.7 ^b^
**C**	208.6 ± 44.5 ^a^	2334.0 ± 73.4 ^a^	2256.4 ± 217.0 ^c^

HT_s_, samples enriched with synthetic Hydroxytyrosol; HT_o_, samples enriched with organic Hydroxytyrosol; C, samples enriched with citric; ^a^, ^b^, ^c^ different letters among data in the same column indicate significant differences among samples (*p* < 0.05).

## Data Availability

Not applicable.
